# Otolaryngology Simulation Curriculum Development and Evaluation for Medical Education in Rwanda

**DOI:** 10.1002/oto2.155

**Published:** 2024-10-24

**Authors:** Sarah Nuss, Rachel Wittenberg, Valerie Salano, Ivy Maina, Gratien Tuyishimire, Mary Jue Xu, Ornella Masimbi, Natnael Shimelash

**Affiliations:** ^1^ Harvard Program in Global Surgery and Social Change Boston USA; ^2^ Warren Alpert Medical School at Brown University Providence Rhode Island USA; ^3^ Harvard Medical School Boston USA; ^4^ Nyahururu County Hospital Nyahururu Kenya; ^5^ Department of Otorhinolaryngology, Penn Medicine Philadelphia USA; ^6^ Department of Surgery/MBC Hospital Kigali Rwanda; ^7^ Department of Otolaryngology–Head and Neck Surgery University of California San Francisco; National Clinician Scholars Program San Francisco USA; ^8^ School of Medicine University of Global Health Equity Butaro Rwanda

**Keywords:** medical education, otolaryngology, simulation

## Abstract

**Objective:**

This study aimed to assess the feasibility and acceptability of a new low‐cost otolaryngology simulation training curriculum for medical students in Rwanda. Given the limited access to hands‐on training and equipment in low‐middle‐income countries, building confidence in performing basic otolaryngology skills is vital for all medical students, especially where all graduates initially serve in primary care before specializing.

**Study Design:**

Preintervention and postintervention assessments of simulation training.

**Setting:**

Conducted at the University of Global Health Equity in Rwanda.

**Methods:**

The simulation program comprised 3 primary components: (1) a low‐cost, moderate‐fidelity model for cricothyrotomy and tracheostomy practice, (2) a low‐cost, low‐fidelity ear model for foreign body and cerumen removal, and a high‐fidelity manikin for practicing, (3) epistaxis management, and (4) nasal foreign body removal. Students underwent pretest and posttest assessments measuring their knowledge, experience, perceived skill, and confidence in performing these procedures. A survey collected feedback on the program.

**Results:**

A total of 29 medical students participated in the simulation program, integrated into a 1‐week otolaryngology “boot camp” preceding a 3‐week clerkship rotation. All models were created using basic, locally available materials, at a total cost of $1.02 for cricothyrotomy and $0.20 for foreign body models. Knowledge and perceived confidence increased for all 3 simulations. All students found the simulations useful, enjoyable, and anticipated using these skills in future training.

**Conclusion:**

The study's results demonstrated that the low‐cost otolaryngology simulation was well‐received and enhanced knowledge, interest, and confidence in performing basic otolaryngology skills across all simulations.

Educational simulation is a valuable tool that can be leveraged for training in skills relevant to head and neck diseases, particularly in low and middle‐income countries (LMIC). Compared to high‐income countries, LMICs carry the greatest burden of otolaryngology–head and neck surgery (OHNS) disease coupled with limited access to subspecialists. However, low‐income countries compared to high‐income countries (HIC) have 50‐fold lower density of OHNS providers.^1^ As a result, the primary care workforce provides a critical portion of care and triaging of head and neck diseases in LMICs. Furthermore, in many LMICs, graduating medical students are required to practice as primary health care providers for a period before pursuing subspecialty training. Implementation of simulation‐based learning for medical students who proceed to practice as part of the primary care workforce in LMICs is therefore invaluable in increasing exposure and preparedness for initial management of OHNS conditions.

While many OHNS simulations currently are resource‐intensive and developed in HIC settings, there is increasing interest in low‐cost models and models relevant to the primary care workforce. Studies have demonstrated that low‐cost and low‐fidelity models can be equally effective in improving performance compared to high‐fidelity simulation models.^2^, ^3^, ^4^, ^5^ In a scoping review by Alayande et al simulation for undergraduate surgical education in Sub‐Saharan Africa, only 25% of programs identified applied simulation based learning in undergraduate training.^6^ Furthermore, these simulations taught up to only 44% of primary care and 32% of first‐level hospital essential surgical procedures as defined by the Disease Control Priority Program (DCP3). And while many models focus on skills relevant to more advanced OHNS training such as cleft lip repair and endoscopic sinus surgery, simulation models for cricothyrotomy, epistaxis management, and ear foreign body removal, for example, are relevant to a broader healthcare workforce.^7^ Given the current limited number of studies assessing the efficacy and sustainability of low‐cost low‐fidelity models in improving performance and confidence, there is a need to develop and evaluate the feasibility, affordability, and acceptability of low‐cost simulation models for trainees entering the primary health care workforce.

A feasibility study of low‐cost simulations for ubiquitous OHNS conditions was performed at the University of Global Health Equity in Rwanda as part of the medical student OHNS rotation. Simulations of clinical scenarios commonly encountered by general practitioners were a core component of the education material delivered, which is highlighted here. Preeducational and posteducational intervention survey assessments investigated the feasibility and student perception of 4 low‐cost simulation models for essential or emergent OHNS conditions for undergraduate medical training in Rwanda.

## Methods

### Study Design

This study involved implementation of OHNS simulations accompanied by presimulation and postsimulation surveys. The study was approved by the institutional review board of the University of Global Health Equity in Rwanda (IRB: UGHE‐IRB/2023/046).

### Study Setting

The University of Global Health Equity is a university located in Butaro, the Northern province of Rwanda. The university has various programs, including the School of Medicine, which employs a rich social medicine and place‐based medical education model. Medical students spend 6 and a half years in training, which includes subspecialty clinical rotations in their 4th year. The OHNS rotation consists of a week of lectures, clinical case discussions, and simulation modules followed by hospital‐based rotations. Upon graduation from medical school, all graduates work as general practitioners in district hospitals around Rwanda for 5 years before considering pursuit of subspeciality training.

### Simulation Development

Four skills were identified as key procedures for management of otolaryngology conditions that would be appropriate for medical students who proceed to practice as general practitioner. These procedures were identified through 2 Delphi studies. The first was a Delphi study conducted at UGHE to identify the most essential procedures for medical students to see during undergraduate training.^8^ The second was a study to identify priority OHNS conditions that all national health systems should be able to care for.^9^ Four simulated skills were identified by the overlap of these 2 lists. The procedures selected were establishing an airway through cricothyrotomy or tracheostomy, ear foreign body removal, epistaxis management, and nasal foreign body removal.

**Figure 1:**
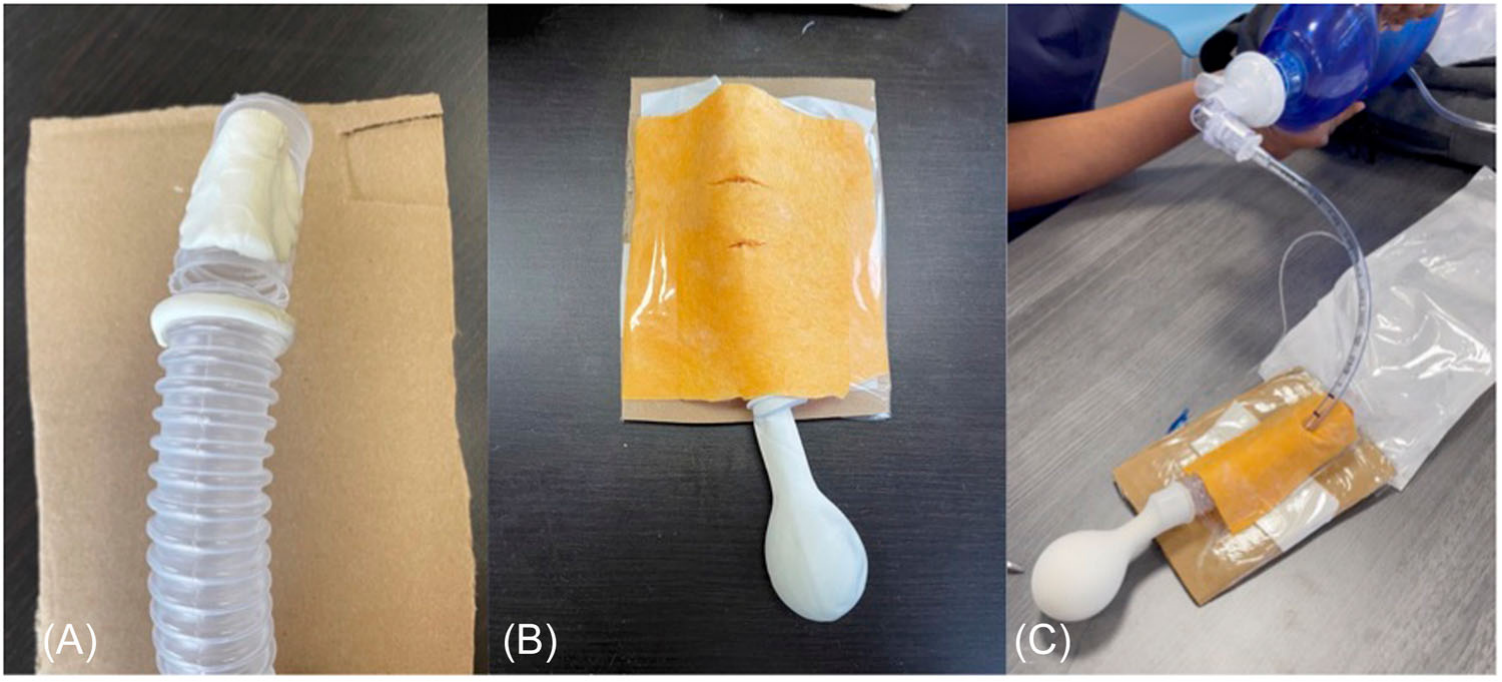
Cricothyrotomy simulation. (A) Ventilation tube with clay cricoid and thyroid cartilage. (B) Completed model. (C) Postcricothyrotomy with endotracheal tube insertion and ventilation through bag‐valve mask. Materials: Ventilation tube, clay, felt, tape, scalpel, endotracheal tube, balloon, bag‐valve mask and bag.

**Figure 2:**
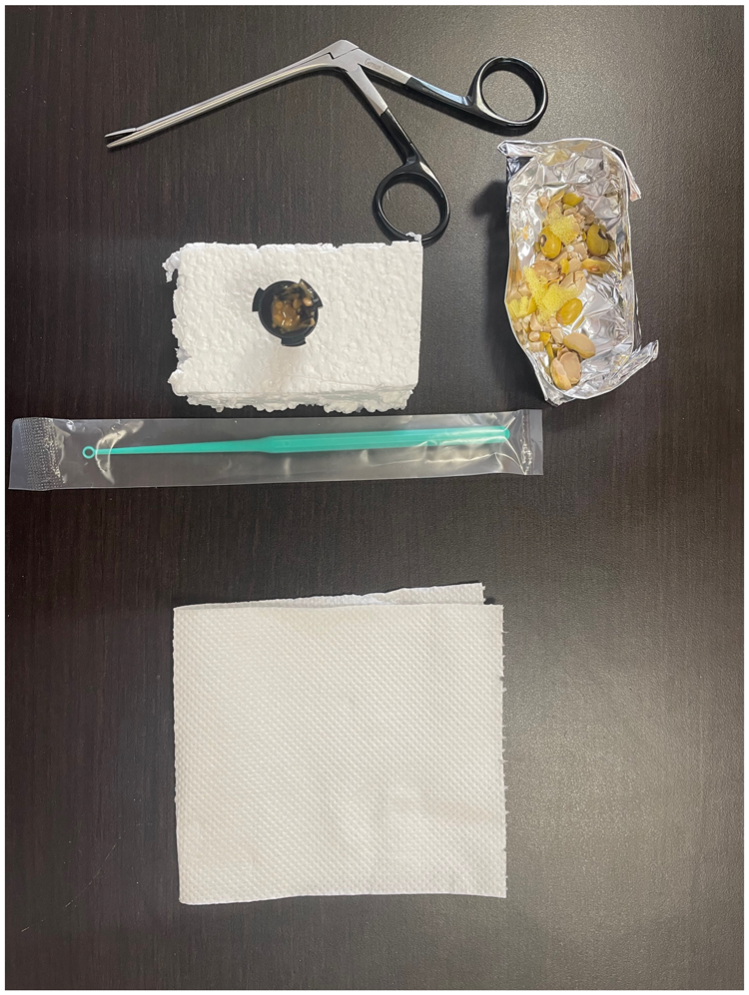
Ear foreign body and cerumen removal. Materials: Otoscope specula, latex glove, curettes, peanut butter, seeds, beans, alligators.

**Figure 3:**
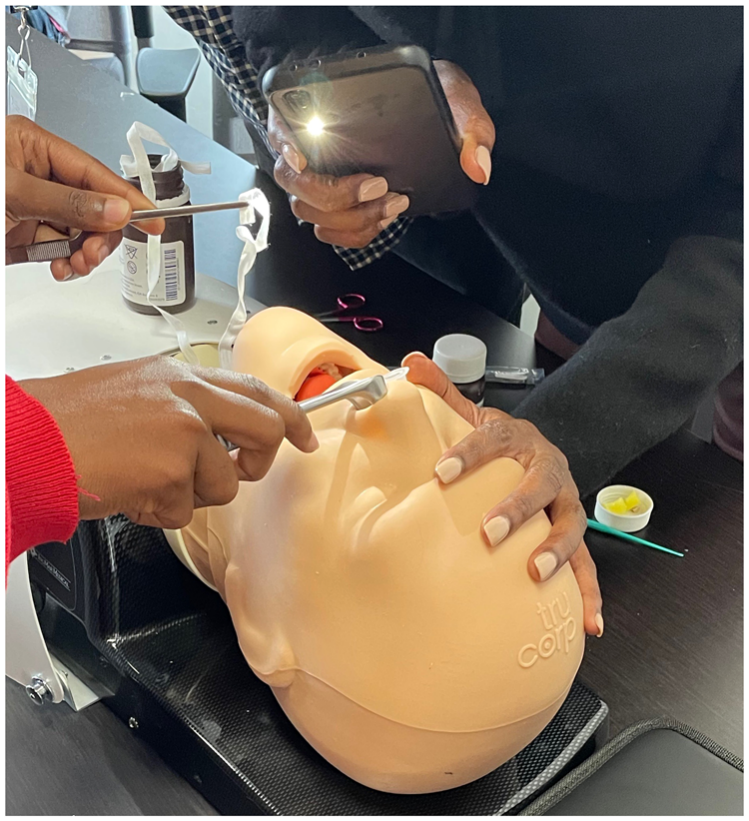
Nasal foreign body removal and epistaxis. Materials: Mannequin head, nasal speculum, gauze, bayonet.

A scoping literature review was conducted to identify examples of low‐cost models for these 4 skills.^10^ Based on the scoping review and materials available locally, the study team worked with the simulation lab at UGHE to develop the following 3 simulation models:

(1) a low‐cost, moderate‐fidelity cricothyrotomy and tracheostomy model (Figure 1).
(2) a low‐cost, low‐fidelity ear model for foreign body and cerumen removal (Figure 2).
(3) a high‐fidelity manikin used for skills sessions of epistaxis management and nasal foreign body removal (Figure 3).

### Model Design

Cricothyrotomy: The cricothyrotomy model was constructed using materials such as clay, felt, ventilation tubing, medical tape, and a balloon. Each model featured a 6″ ventilation tube mounted on a piece of cardboard backing, with a section removed from the front. Clay pieces fashioned into cricoid and thyroid cartilage were positioned below and above the gap in the ventilation tube, while medical tape simulated a cricothyroid membrane. A balloon was affixed to the bottom of the ventilation tube, and a layer of felt cloth secured over the front mimicked skin.

Ear cerumen/foreign body removal: For the ear cerumen/foreign body removal model, materials included 2 otoscope tips per model, latex gloves, peanut butter, sesame seeds, and beans. The otoscope tips were stacked with a small piece of latex between them to represent a tympanic membrane, inserted into Styrofoam for stability, and filled with a mixture of peanut butter, sesame seeds, and beans to mimic ear canal contents.

Nasal foreign body and epistaxis model: The nasal foreign body and epistaxis model utilized a high‐fidelity mannequin head equipped with nasal passages, nasal specula, and gauze. Gauze squares were demonstrated for converting into strip gauze as part of the model's instructional utility.

### Simulation Implementation

The simulations developed were incorporated into an intensive week of lectures and case discussions before hospital‐based clinical rotations. The sessions were accompanied by a short lecture or case‐based discussion on the topic related to the simulation.

### Data Collection

Twenty‐nine medical students first were evaluated with a presimulation survey gauging content knowledge, experience, perceived skill, and confidence in performing the procedures (Supplemental Appendix S1, available online). Following each simulation module, a postsimulation survey focused on content knowledge, confidence, perceived utility, and enjoyment of the simulation was delivered (Supplemental Appendix S1, available online).

### Data Analysis

Descriptive analyses were used to summarize presimulation and postsimulation responses.

## Results

### Demographics of Participants and Model Information

A total of 29 medical students participated in the simulation program, with each model taking less than 5 minutes to construct per model. All materials used for the models were locally available, except for alligators and ear curettes. Tools such as nasal speculums (estimated cost $50.00 per speculum), ear curettes (estimated cost $15 per curette), and alligators (estimated cost $30 per forceps) were donated and reusable and not included in the cost calculation for the models. The total cost for the simulation models was $1.02 for each cricothyrotomy/tracheostomy model and $0.20 per foreign body model. The overall expenditure for all 29 students in utilizing these simulation models amounted to $45.44 (Table 1).

**Table 1:** Materials and Costs of Models

Material	Cost per unit (USD)	Cost per 29 students
**Cricothyrotomy**		
Clay	0.07	2.03
Felt	0.17	4.93
Ventilator tube	0.50	14.5
Clear tape	0.07	2.03
Cloth tape	0.12	3.48
Balloon	0.09	2.61
***Total***	1.02	29.53
**Ear foreign body/cerumen removal**		
Otoscope tips	0.18	5.22
Gloves	0.01	0.29
Peanut butter	0.01	0.29
Sesame seeds	0.01	0.29
Styrofoam	0.00	0.00
***Total***	0.20	5.88
**Nasal model (epistaxis + foreign body)**		
Mannequin head	Donated	
Nasal specula	Donated	
Gauze	0.35	10.03
***Total***	0.35	10.03
**Total cost**	1.57	**45.44**

### Simulation Curriculum Feasibility

Participants reported a substantial increase in their perceived confidence across all 4 simulations. For all simulations, over 50% of the students had no prior exposure to these skills, with over 90% having no prior exposure for all simulations except for epistaxis. Participants expressed a high level of satisfaction with the simulation models, with more than 95% finding them useful (agree or strongly agree) across all simulations. Additionally, the models were deemed to be somewhat or very representative of real‐world scenarios by over 80% of the participants. Furthermore, 100% agreed or strongly agreed that they enjoyed the simulation session. A significant majority of participants (>95% across all models) indicated their anticipation of applying the skills acquired during these simulations in their future clinical practice. A summary of these results is detailed in Table 2.

**Table 2:** Student Pre/Postsimulation Assessment Results

	Epistaxis	Ear foreign body	Cricothyrotomy	Nasal foreign body
**Interest**				
*How interested are you in learning this skill?*				
*Very interested*	100%	86%	86%	100%
*Somewhat interested*	0%	0%	0%	0%
*Neutral*	0%	3%	4%	0%
*Somewhat uninterested*	0%	10%	10%	0%
*Very uninterested*	0%	0%	0%	0%
**Utility**				
*Anticipate using skill in future*	100%	100%	95%	100%
*Found it very useful*	93%	93%	100%	97%
*Enjoyed session*	100%	97%	100%	100%
**Knowledge**				
*Average preassessment score*	55.9%	80.7%	85.6%	58.1%
*Average postassessment score*	90.9%	77.4%	88.9%	70.3%
*Increase in average score*	35.0%	3.3%	12.3%	−3.2%
**Confidence**				
*Increase in confidence score (% students)*	84%	90%	86%	80%
**Representative**				
*Somewhat representative*	65%	85%	100%	91%
*Very representative*	18%	8%	0%	0%
**Prior exposure**				
*Never*	55%	90%	100%	90%

## Discussion

In this study, we introduced low‐cost OHNS simulations aimed at medical students in Rwanda who will practice in the primary healthcare workforce following graduation. We found that implementing a simulation curriculum in this setting was feasible, raised learner confidence, and were able to contain costs to <$2.00 per model. All supplies needed for these simulations were locally available except for some reusable instruments that were donated to the school. The cost for 29 learners for the 4 models—cricothyrotomy, nasal foreign body removal, epistaxis, and ear foreign body/cerumen removal—were $45.44 total. These models and assessments can be readily adapted for use in in other resource‐constrained health setting to facilitate the training of primary healthcare professionals in the management of commonly encountered and emergent OHNS conditions.

There is a need for increased development and focus on simulations for a primary healthcare workforce. A recent scoping review conducted to identify such models found few OHNS models designed to address the needs of general practitioners (GPs), who potentially would benefit the most from low‐cost OHNS simulations (under review). Furthermore, the study identified a need for models to be developed by or in collaboration with LMIC institutions to enhance their accessibility and sustainability. More standardized, objective evaluations should also be used to gauge the efficacy of these models in enhancing skill performance, confidence, and knowledge. Given that OHNS care and management emphasizes procedures, simulation is critical component to incorporate into training. Given the procedural nature of OHNS care and management, the integration of simulation into training is pivotal. For instance, the World Health Organization Primary Ear and Hearing Care Curriculum integrates skills such as otoscopy into its program tailored for primary care providers.^11^ The inclusion of low‐cost simulations has the potential to enrich and augment such curricula.

In addition to a focus on graduating medical students, these simulations have broader applicability to the existing practicing primary care workforce and be incorporated into continuing medical education curriculum both in LMICs and HICs. Continuing medical education (CME) is a requirement in many countries such as United States, United Kingdom, China, Canada, Australia, Indonesia, and other European countries.^12^, ^13^ For example, in China, 100% of senior, 95% of midgrade, and 80% of junior health professionals are required to update their training through CME.^14^ Traditionally, CME has been completed through didactics and online training courses, however, there is increased interest in incorporating simulation as a CME modality to keep up with the worldwide shift towards more experiential learning.^15^, ^16^ Given the limited time to teach otolaryngology during undergraduate medical education and the low lack of opportunity to develop and practice skills amongst primary care workers, there is an opportunity to incorporate OHNS simulation into CME into these curricula.^17^, ^18^, ^19^, ^20^

The implications of this study extend beyond our immediate findings and demonstrate promising opportunities for future low‐cost simulation in OHNS care. The decreasing cost of cutting‐edge technologies such as virtual reality and 3‐dimensional printing opens doors for their integration in OHNS simulation‐based training in low‐resourced settings. As these technologies become more accessible, they hold the potential to further enhance the realism and efficacy of training. Virtual training modules could also help overcome geographic barriers to increase accessibility of simulation‐based training to rural and remote settings. Additionally, these simulation models can be adapted to enable task shifting by training of healthcare professionals in procedures that may not be in their current scope of practice, such as peritonsillar abscess aspiration. Finally, the application of these simulations across different specialties, from Emergency Medicine to Family Medicine, encourages interdisciplinary training and collaboration, better‐preparing providers for team‐based healthcare delivery.

### Limitations

The primary limitation to this study is that simulations were assessed subjectively. While subjective assessment of acceptability provide valuable insights, their reliance on personal judgement may not adequately gauge the actual proficiency of participants.^21^ To comprehensively evaluate the efficacy of the curriculum, it is imperative to incorporate objective skill metrics and conduct continuous assessments of skill retention and knowledge. Additionally, 80% of participants reported that models simulated real‐world circumstances, yet most of the participants have not yet had significant clinical experience. Future simulation sessions should be evaluated by experienced general practitioners or otolaryngologists within Rwanda to evaluate the model's representativeness of context‐specific clinical experiences. Furthermore, our study is limited by the small sample size within the simulation curriculum at UGHE. This limitation can be addressed through future research involving additional cohorts of students. In addition, our study used primarily locally sourced materials; however, some materials such as surgical instruments and mannequin heads were donated or not readily available in many LMIC settings. To ensure sustainability of these models, future adaptations to minimize reliance on external procurement is necessary. Finally, it is worth noting that certain key skills such as ear irrigation, incision and drainage of superficial abscess, and facial lacerations were not captured by our method to identify key skills for simulation. Future studies should seek to develop and evaluate low‐cost models for additional OHNS skills, particularly those relevant to GPs.

## Conclusion

Simulation of critical skills for management of OHNS conditions by primary care providers can be incorporated into curriculum in resource‐constrained health settings in a feasible, acceptable, and low‐cost manner. Future studies will be strengthened by objective evaluation and a focus on skill retention of practicing primary care providers.

## Author Contributions

**Sarah Nuss**, study design, data collection, data analysis, manuscript writing, manuscript revision, final approval of manuscript; **Rachel Wittenberg**, study design, data collection, data analysis, manuscript revision, final approval of manuscript; **Valerie Salano**, study design, data interpretation, manuscript revision, final approval of manuscript; **Ivy Maina**, study design, data collection, manuscript revision, final approval of manuscript; **Gratien Tuyishimire**, study design, data interpretation, manuscript revision, final approval of manuscript; **Mary Jue Xu**, study design, data interpretation, manuscript revision, final approval of manuscript; **Ornella Masimbi**, study conception, project oversight, study design, manuscript revision, final approval of manuscript; **Natnael Shimelash**, study conception, project oversight, study design, manuscript revision, final approval of manuscript.

## Disclosure

### Competing interests

None.

### Funding source

SRN is supported by the Fogarty International Center and National Institute of Mental Health, of the National Institutes of Health under Award Number D43 TW010543. This study is a part the study funded by NIH grant number NIH/FIC‐ 5R21HD103052‐02. The content is solely the responsibility of the authors and does not necessarily represent the official views of the National Institutes of Health.

## Supporting information

Supporting information.

## References

[1] Petrucci B , Okerosi S , Patterson RH , et al. The global otolaryngology‐head and neck surgery workforce. JAMA Otolaryngol Head Neck Surg. 2023;31:e232339. 10.1001/jamaoto.2023.2339 PMC10472262 37651133

[2] Nicolaides M , Theodorou E , Emin EI , et al. Team performance training for medical students: low vs high fidelity simulation. Ann Med Surg. 2020;55:308‐315. 10.1016/j.amsu.2020.05.042 PMC7292889 32551104

[3] Munshi F , Lababidi H , Alyousef S . Low‐ versus high‐fidelity simulations in teaching and assessing clinical skills. J Taibah Univer Med Sci. 2015;10(1):12‐15. 10.1016/j.jtumed.2015.01.008

[4] De Giovanni D , Roberts T , Norman G . Relative effectiveness of high‐ versus low‐fidelity simulation in learning heart sounds. Med Educ. 2009;43(7):661‐668. 10.1111/j.1365-2923.2009.03398.x 19573189

[5] Lee KHK , Grantham H , Boyd R . Comparison of high‐ and low‐fidelity mannequins for clinical performance assessment. Emerg Med Australas. 2008;20(6):508‐514. 10.1111/j.1742-6723.2008.01137.x 19125830

[6] Alayande B , Forbes C , Masimbi O , et al. The implementation of simulation‐based learning for training udergraduate Medical Students in essential surgical care across sub‐saharan Africa: a scoping review. Med Sci Educ. 2023;34(1):237‐256. 10.1007/s40670-023-01898-6 38510415 PMC10948665

[7] Pankhania R , Pelly T , Bowyer H , Shanmugathas N , Wali A . A systematic review of low‐cost simulators in ENT surgery. J Laryngol Otol. 2021;135(6):486‐491. 10.1017/S0022215121000839 33734059

[8] Alayande BT , Iradukunda J , Forbes CW , et al. Using a modified Delphi process to identify critical topics for junior and senior surgery clerkships in the rural Rwandan context. J Am Coll Surg. 2022;235(5):S236. 10.1097/01.XCS.0000894856.54724.c2

[9] Nuss S , Patterson RH , Cahill GL , et al. Delphi method consensus on priority global otolaryngology–head and neck surgery conditions and procedures. Otolaryngol Neck Surg. 2022;25:019459982110737. 10.1177/01945998211073705 35077240

[10] Nzisabira J , Nuss S , Candelo E , et al. Low‐cost otolaryngology simulation models for early‐stage trainees: a scoping review. BMC Med Educ. 2024;24:483. 10.1186/s12909-024-05466-3 38693491 PMC11062898

[11] World Health Organization (WHO) . *Primary Ear and Hearing Care Manual*. World Health Organization. 2023. https://www.who.int/teams/noncommunicable-diseases/sensory-functions-disability-and-rehabilitation/primary-ear-and-hearing-care-training-manual

[12] Institute of Medicine (US) Committee on Planning a Continuing Health Professional Education Institute . International Comparison of Continuing Education and Continuing Professional Development. Redesigning Continuing Education in the Health Professions. National Academies Press (US); 2010:2023. https://www.ncbi.nlm.nih.gov/books/NBK219811

[13] Miller LA , Chen X , Srivastava V , Sullivan L , Yang W , Yii C . CME credit systems in three developing countries: China, India, and Indonesia. J Eur CME. 2015;4(1):27411. 10.3402/jecme.v4.27411

[14] Wong WCW , Zhu S , Ong JJ , et al. Primary care workforce and continuous medical education in China: lessons to learn from a nationwide cross‐sectional survey. BMJ Open. 2017;7:e015145. 10.1136/bmjopen-2016-015145 PMC5734561 28710208

[15] Datta R , Upadhyay K , Jaideep C . Simulation and its role in medical education. Med J Armed Forces India. 2012;68(2):167‐172. 10.1016/S0377-1237(12)60040-9 24623932 PMC3862660

[16] Khanduja PK , Bould MD , Naik VN , Hladkowicz E , Boet S . The Role of simulation in continuing medical education for acute care physicians: a systematic review. Crit Care Med. 2015;43(1):186‐193. 10.1097/CCM.0000000000000672 25343571

[17] Hu A , Sardesai MG , Meyer TK . A need for otolaryngology education among primary care providers. Med Educ Online. 2012;17(1):17350. 10.3402/meo.v17i0.17350 22754276 PMC3386554

[18] Baba SH . Community ENT Health Promotion: Concepts and Perspectives in Mali. Int Res J Pharm Med Sci. 2023;6(2):38‐40.

[19] Wong A , Fung K . Otolaryngology in undergraduate medical education. J Otolaryngol Head Neck Surg. 2009;38(1):38‐48. 19344612

[20] Carr MM , Brown DH , Reznick RK . Needs assessment for an undergraduate otolaryngology curriculum. Otolaryngol Head Neck Surg. 1999;120(6):865‐868. 10.1016/S0194-5998(99)70328-1 10352441

[21] Bewley WL , O'Neil HF . Evaluation of medical simulations. Mil Med. 2013;178(suppl_10):64‐75. 10.7205/MILMED-D-13-00255 24084307

