# Supplementary material for: Otolaryngology Simulation Curriculum Development and Evaluation for Medical Education in Rwanda
Source: OTO Open. 2024 Oct 24;8(4):e155. doi: 10.1002/oto2.155 (PMC11499706; doi:10.1002/oto2.155)
Supplement: Supplementary file 1 — Supporting information. [file OTO2-8-e155-s001.docx]

**Appendix 1**

**Cricothyrotomy Pre-Assessment**

Identification Number:

Which of the following do you cut through in a cricothyrotomy?

A. Thyrohyoid membrane

B. Cricothyroid membrane

C. Cricoid cartilage

D. All of the above

When is it appropriate to consider doing a surgical cricothyrotomy?

A. Cannot intubate

B. Cannot oxygenate

C. Children under 12 with airway obstruction

D. Both A and B

E. All of the above

Which of the following is the least required to perform a cricothyrotomy and obtain a stable airway?

A. Scalpel

B. Airway tubing

C. Suction

D. Ventilator

A needle cricothyrotomy is preferred for children under the age of 12

A. True

B. False

The incision for a cricothyrotomy is

A. Horizontal

B. Vertical

C. It doesn’t matter

**Experience**

Have you observed an emergency cricothyrotomy in a clinical setting?

A. Yes, many times

B. Yes, a few times

C. Yes, once

D. No, never

E. I’m not sure

**Interest**

How interested are you in learning to perform an emergency cricothyrotomy?

A. Very interested

B. Somewhat interested

C. Neutral

D. Somewhat uninterested

E. Very uninterested

**Skill**

A. How confident do you feel in your skills to perform an emergency cricothyrotomy?

B. Very confident

C. Somewhat confident

D. Neutral

E. Somewhat un-confident

F. Not confident at all

**Epistaxis and Nasal Foreign Body Pre-Assessment**

Identification Number:

**Knowledge**

Which of the following is NOT a step in managing epistaxis?

A. Lean back / tilt your head back

B. Pinch your nose

C. Nasal congestion spray (oxymetazoline)

Where is the most common origin of epistaxis?

A. Anterior nasal septum

B. Anterior inferior turbinate

C. Posterior nasal septum

D. Choana

A patient comes in with epistaxis. What is your initial step in management?

A. Evaluate airway

B. Order an FBC

C. Take vitals

D. Consult ENT

E. Pack the nose

A patient recent had epistaxis and nasal packing. They return with fever, hypotension, and vomiting. What is the most likely diagnosis?

A. Strep infection

B. Anaphylactic reaction to packing

C. Toxic Shock Syndrome

D. Hemorrhagic shock

True or False: You should give an intranasal decongestant (oxymetazoline) to help manage epistaxis

A. True

B. False

Which of the following would be the preferred material to pack a posterior nosebleed?

A. Regular gauze

B. Strip gauze (continuous)

C. Cotton balls

D. You shouldn’t pack the nosebleed

Which of the following is a reason to refer a nasal foreign body patient to a tertiary center?

A. Very posterior foreign body

B. Very small foreign body

C. Multiple unsuccessful attempts to remove foreign body

D. Improper/difficult visualization

E. A and B

F. C and D

G. All of the above

True or False: Nasal foreign body removal without proper visualization can cause inhalation of the foreign body.

A. True

B. False

**Experience**

Have you observed epistaxis management in a clinical setting?

A. Yes, many times

B. Yes, a few times

C. Yes, once

D. No, never

E. I’m not sure

Have you observed nasal foreign body removal in a clinical setting?

A. Yes, many times

B. Yes, a few times

C. Yes, once

D. No, never

E. I’m not sure

**Interest**

How interested are you in learning to manage epistaxis?

A. Very interested

B. Somewhat interested

C. Neutral

D. Somewhat uninterested

E. Very uninterested

How interested are you in learning nasal foreign body removal?

A. Very interested

B. Somewhat interested

C. Neutral

D. Somewhat uninterested

E. Very uninterested

**Skill**

How confident do you feel in your skills to manage epistaxis?

A. Very confident

B. Somewhat confident

C. Neutral

D. Somewhat un-confident

E. Not confident at all

How confident do you feel in your skills to manage a nasal foreign body (remove or refer when appropriate)?

A. Very confident

B. Somewhat confident

C. Neutral

D. Somewhat un-confident

E. Not confident at all

**Ear Foreign Body Pre-Assessment**

Identification Number:

**Knowledge**

What is the best technique for removing an insect in the ear that is obstructing the tympanic membrane?

A. Irrigation

B. Manual extraction

C. Q-tip and glue

What are needed to perform a safe foreign body removal?

A. Instruments (forceps or curette)

B. Illumination

C. Still/Non-moving patient

D. All of the above

If you have impacted cerumen that you cannot remove on initial attempt using a curette, what should you do?

A. Continue another attempt using the curette

B. Ear syringing

C. Give ear drops to soften the wax for removal at a later date

D. Use a q-tip to remove the wax

E. After section 2

**Experience**

Have you observed an ear foreign body removal in a clinical setting?

A. Yes, many times

B. Yes, a few times

C. Yes, once

D. No, never

E. I’m not sure

**Interest**

How interested are you in learning ear foreign body removal?

A. Very interested

B. Somewhat interested

C. Neutral

D. Somewhat uninterested

E. Very uninterested

**Skill**

How confident do you feel in your skills to perform an ear foreign body removal?

A. Very confident

B. Somewhat confident

C. Neutral

D. Somewhat un-confident

E. Not confident at all

**Cricothyrotomy Post-Assessment**

Identification Number

**Knowledge**

Which of the following do you cut through in a cricothyrotomy?

A. Thyrohyoid membrane

B. Cricothyroid membrane

C. Cricoid cartilage

D. All of the above

When is it appropriate to consider doing a surgical cricothyrotomy?

Cannot intubate

A. Cannot oxygenate

B. Children under 12 with airway obstruction

C. Both A and B

D. All of the above

Which of the following is the least required to perform a cricothyrotomy and obtain a stable airway?

Scalpel

A. Airway tubing

B. Suction

C. Ventilator

A needle cricothyrotomy is preferred for children under the age of 12

A. True

B. False

The incision for a cricothyrotomy is

A. Horizontal

B. Vertical

C. It doesn’t matter

**Experience**

Have you observed an emergency cricothyrotomy in a clinical setting?

A. Yes, many times

B. Yes, a few times

C. Yes, once

D. No, never

E. I’m not sure

**Interest**

How interested are you in learning to perform an emergency cricothyrotomy?

A. Very interested

B. Somewhat interested

C. Neutral

D. Somewhat uninterested

E. Very uninterested

**Skill**

How confident do you feel in your skills to perform an emergency cricothyrotomy?

A. Very confident

B. Somewhat confident

C. Neutral

D. Somewhat un-confident

E. Not confident at all

**Simulation Feedback**

I found this simulation very useful/worthwhile

A. Strongly

B. Agree

C. Neutral

D. Disagree

E. Strongly Disagree

If you have observed or practiced an emergency cricothyrotomy in a clinical setting, how representative do you feel the model was:

A. Very representative

B. Somewhat representative

C. Neither representative nor unrealistic

D. Somewhat unrealistic

E. Very unrealistic

F. I have not observed this skill in a clinical setting

I anticipate using this skill in my future training and practice

A. Strongly agree

B. Strongly disagree

C. Disagree

D. Neutral

E. Agree

I enjoyed this session

A. Strongly Agree

B. Agree

C. Neutral

D. Disagree

E. Strongly Disagree

**Ear Foreign Body Post-Assessment**

Identification Number:

**Knowledge**

What is the best technique for removing an insect in the ear that is obstructing the tympanic membrane?

A. Irrigation

B. Manual extraction

C. Q-tip and glue

What materials are needed to perform a safe foreign body removal?

A. Instruments (forceps or curette)

B. Illumination

C. Still/Non-moving patient

D. All of the above

If you have impacted cerumen that you cannot remove on initial attempt using a curette, what should you do?

A. Continue another attempt using the curette

B. Ear syringing

C. Give ear drops to soften the wax for removal at a later date

D. Use a q-tip to remove the wax

**Experience**

Have you observed an ear foreign body removal in a clinical setting?

A. Yes, many times

B. Yes, a few times

C. Yes, once

D. No, never

E. I’m not sure

**Interest**

How interested are you in learning ear foreign body removal?

A. Very interested

B. Somewhat interested

C. Neutral

D. Somewhat uninterested

E. Very uninterested

**Skill**

How confident do you feel in your skills to perform an ear foreign body removal?

A. Very confident

B. Somewhat confident

C. Neutral

D. Somewhat un-confident

E. Not confident at all

**Simulation Feedback**

I found this simulation very useful/worthwhile

A. Strongly

B. Agree

C. Neutral

D. Disagree

E. Strongly Disagree

If you have observed or practiced an ear foreign body removal in a clinical setting, how representative do you feel the model was:

A. Very representative

B. Somewhat representative

C. Neither representative nor unrealistic

D. Somewhat unrealistic

E. Very unrealistic

F. I have not observed this skill in a clinical setting

I anticipate using this skill in my future training and practice

A. Strongly agree

B. Strongly disagree

C. Disagree

D. Neutral

E. Agree

I enjoyed this session

A. Strongly Agree

B. Agree

C. Neutral

D. Disagree

E. Strongly Disagree

**Epistaxis and Nasal Foreign Body Post-Assessment**

Identification Number:

**Knowledge**

Which of the following is NOT a step in managing epistaxis?

A. Lean back / tilt your head back

B. Pinch your nose

C. Nasal congestion spray (oxymetazoline)

Where is the most common origin of epistaxis?

A. Anterior nasal septum

B. Anterior inferior turbinate

C. Posterior nasal septum

D. Choana

A patient comes in with epistaxis. What is your initial step in management?

A. Evaluate airway

B. Order an FBC

C. Take vitals

D. Consult ENT

E. Pack the nose

A patient recent had epistaxis and nasal packing. They return with fever, hypotension, and vomiting. What is the most likely diagnosis?

A. Strep infection

B. Anaphylactic reaction to packing

C. Toxic Shock Syndrome

D. Hemorrhagic shock

True or False: You should give an intranasal decongestant (oxymetazoline) to help manage epistaxis

A. True

B. False

Which of the following would be the preferred material to pack a posterior nosebleed?

Regular gauze

A. Strip gauze (continuous)

B. Cotton balls

C. You shouldn’t pack the nosebleed

Which of the following is a reason to refer a nasal foreign body patient to a tertiary center?

A. Very posterior foreign body

B. Very small foreign body

C. Multiple unsuccessful attempts to remove foreign body

D. Improper/difficult visualization

E. A and B

F. C and D

G. All of the above

True or False: Nasal foreign body removal without proper visualization can cause inhalation of the foreign body.

A. True

B. False

**Experience**

Have you observed epistaxis management in a clinical setting?

A. Yes, many times

B. Yes, a few times

C. Yes, once

D. No, never

E. I’m not sure

Have you observed nasal foreign body removal in a clinical setting?

Yes, many times

Yes, a few times

Yes, once

No, never

I’m not sure

**Interest**

How interested are you in learning to manage epistaxis?

A. Very interested

B. Somewhat interested

C. Neutral

D. Somewhat uninterested

E. Very uninterested

How interested are you in learning nasal foreign body removal?

A. Very interested

B. Somewhat interested

C. Neutral

D. Somewhat uninterested

E. Very uninterested

**Skill**

How confident do you feel in your skills to manage epistaxis?

A. Very confident

B. Somewhat confident

C. Neutral

D. Somewhat un-confident

E. Not confident at all

How confident do you feel in your skills to manage a nasal foreign body (remove or refer when appropriate)?

A. Very confident

B. Somewhat confident

C. Neutral

D. Somewhat un-confident

E. Not confident at all

**Simulation Feedback**

I found this simulation very useful/worthwhile

A. Strongly

B. Agree

C. Neutral

D. Disagree

E. Strongly Disagree

I found this simulation (epistaxis management) very useful/worthwhile

A. Strongly

B. Agree

C. Neutral

D. Disagree

E. Strongly Disagree

I found this simulation (nasal foreign body removal) very useful/worthwhile

A. Strongly

B. Agree

C. Neutral

D. Disagree

E. Strongly Disagree

If you have observed or practiced epistaxis management in a clinical setting, how representative do you feel the model was:

A. Very representative

B. Somewhat representative

C. Neither representative nor unrealistic

D. Somewhat unrealistic

E. Very unrealistic

F. I have not observed this skill in a clinical setting

If you have observed or practiced nasal foreign body removal in a clinical setting, how representative do you feel the model was:

A. Very representative

B. Somewhat representative

C. Neither representative nor unrealistic

D. Somewhat unrealistic

E. Very unrealistic

F. I have not observed this skill in a clinical setting

I anticipate using this skill (epistaxis management) in my future training and practice

A. Strongly agree

B. Strongly disagree

C. Disagree

D. Neutral

E. Agree

I anticipate using this skill (nasal foreign body removal) in my future training and practice

A. Strongly agree

B. Strongly disagree

C. Disagree

D. Neutral

E. Agree

I enjoyed this session

A. Strongly Agree

B. Agree

C. Neutral

D. Disagree

E. Strongly Disagree

Have you had exposure to simulation in your medical education prior to this week?

A. Yes, a lot of exposure

B. Yes, a little exposure

C. No exposure

D. Not sure
